# Marginal Ulcer Perforation in a Whipple Survivor: A Rare Long-Term Complication

**DOI:** 10.7759/cureus.28050

**Published:** 2022-08-16

**Authors:** Arunraj P, Kolandasamy C, Prabhakaran R, Sugumar Chidambaranathan, Naganath Babu O L

**Affiliations:** 1 Surgical Gastroenterology, Madras Medical College, Chennai, IND

**Keywords:** pancreaticoduodenectomy, whipple procedure, proton pump inhibitor, vagotomy, marginal ulcer perforation

## Abstract

Long-term complications following pancreaticoduodenectomy are rarely reported because of the poor long-term survival of these patients. Marginal ulcers can occur in both the short and long term, and they can become complicated by bleeding or perforation. Marginal ulcer with perforation is a rare incidence and only sparse literature evidence is available. Herein, we report on a patient who underwent pancreaticoduodenectomy 12 years ago for duodenal adenocarcinoma and was diagnosed to have perforation peritonitis. He underwent emergency laparotomy and lavage, and omental patch closure for marginal ulcer perforation at the gastrojejunostomy site. Truncal vagotomy and feeding jejunostomy were also done.

## Introduction

Overall, the five-year survival rate for patients with periampullary carcinoma undergoing pancreaticoduodenectomy (PD) is around 20-25%, and a 10-year survival rate of 13% has been reported [1]. This was mainly due to improvements in surgical techniques and postoperative care. Studies published on PD mainly report three major complications, namely, postoperative pancreatic fistula (POPF), delayed gastric emptying (DGE), and post-pancreatectomy hemorrhage [2,3]. Because marginal ulcer is a rare long-term complication, morbidity and mortality associated with it and its complications, namely, bleeding and perforation, are not frequently reported [4]. A marginal ulcer with complications may become fatal if not diagnosed or prevented early. This is our experience of marginal ulcer perforation in a Whipple survivor of 12 years that we encountered at our institute.

## Case presentation

A 57-year-old male patient who underwent the classical Whipple procedure 12 years ago for duodenal adenocarcinoma (well-differentiated adenocarcinoma, papillary type) presented with complaints of diffuse abdominal pain and fever for a one-week duration. He underwent the Whipple procedure 12 years ago and he has been on regular follow-up for five years. No adjuvant treatment was given as the patient denied consent for the same. After five years, he did not come for regular follow-ups. The patient had no other comorbid illnesses.

On examination, the patient was febrile, tachycardia was present, and his blood pressure was 110/70 mmHg. He was also guarding and experiencing diffuse abdominal tenderness. The rectal examination was normal. The hemogram showed leucocytosis and other blood parameters like renal function tests and liver function tests were normal. A contrast-enhanced computed tomography (CECT) of the abdomen was taken, which showed dilated and edematous small bowel loops, and there was no evidence of disease recurrence. With these findings in the background, he was clinically diagnosed with a case of peritonitis, probably due to adhesive small bowel obstruction leading to perforation.

**Figure 1:**
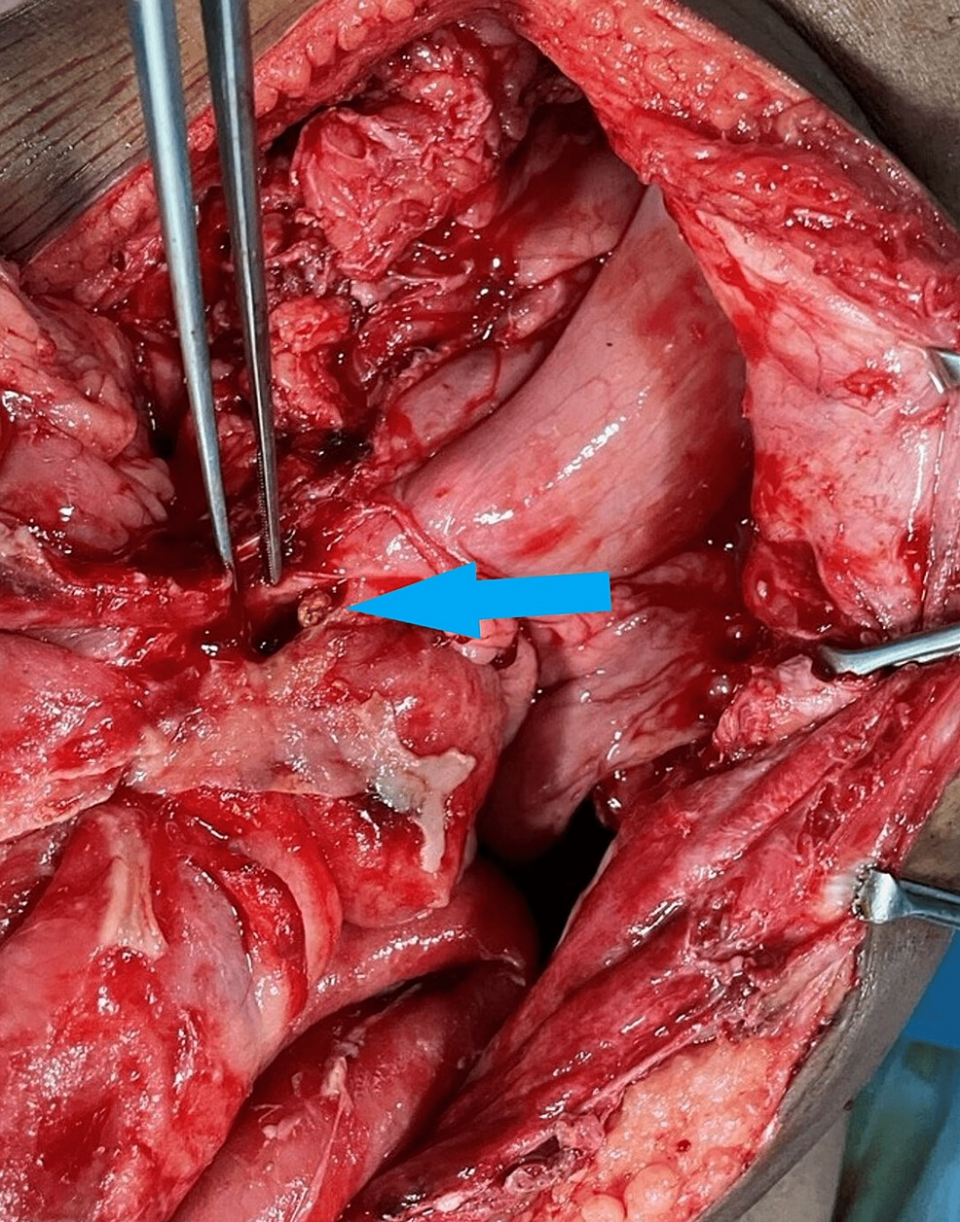
Intraoperative picture showing marginal ulcer perforation

An emergency laparotomy was done. The findings were 500 ml of seropurulent fluid in the peritoneal cavity and dense adhesion between the small bowel loops and previous surgical scar. Adhesions were meticulously released and gastrojejunostomy site perforation was there, which was around 1 cm. A thorough peritoneal lavage was done and the gastrojejunostomy site perforation was closed with a well-vascularized omental patch after a biopsy from the ulcer edge. We also did a bilateral truncal vagotomy and a feeding jejunostomy (Figure 1).

Postoperatively, we were unable to remove the Ryles tube because of high Ryles tube output till postoperative day (POD) 12. Feeding jejunostomy was started on POD two, which the patient tolerated well. Failure of the removal of the Ryles tube was attributed to symptoms akin to delayed gastric emptying. He was managed conservatively until POD 12. The patient was started on an oral diet on POD 12. He recovered well and was discharged on POD 17. Postoperative histopathological examination (HPE) from ulcer biopsy showed no evidence of dysplasia or malignancy.

## Discussion

A marginal ulcer usually develops at or near the gastrojejunal or duodenojejunal anastomosis. The location of the ulcer is within 3 cm of the anastomosis, and it usually occurs on the jejunal side [5]. The incidence of marginal ulceration following PD ranges from 0% to 18% with a mean incidence of 2.5%, and the median time to diagnosis is 15.5 months [6]. Risk factors associated with the development of marginal ulcers are discontinuation of proton pump inhibitor (PPI) therapy (an independent risk factor), smoking, alcohol intake, use of nonsteroidal anti-inflammatory drugs (NSAIDs), and *Helicobacter pylori *infection.

There are several mechanisms proposed for the development of a marginal ulcer. The local effect of postoperative scarring, inflammation, angulation, and tension is thought to be instrumental in the development of a marginal ulcer. The presence of foreign bodies (sutures and staples) is a causative factor in the early postoperative period. The effect of gastric acid on at-risk jejunal mucosa will lead to marginal ulcers in later periods [7].

There was no difference in the incidence of marginal ulcers between procedures when the extent of the gastric resection (pylorus-preserving PD (PPPD) versus pylorus-resecting PD (PRPD) versus classical Whipple) was compared [5]. Between pylorus preservation and "classic" PD, the incidence was 2% versus 2.6%, and there was not much difference. Retained antral mucosa was also described as a cause [8].

Based on the reconstruction technique following PD, there was an increased incidence after Roux-en-Y reconstruction. This may be due to the diversion of duodenal and pancreatic secretions away from the anastomosis, which is rich in bicarbonate (protective by buffering low pH). Additionally, the bicarbonate output may decrease after PD [9-11]. Another factor that may contribute to a higher incidence with Roux-en-Y reconstruction is a lower incidence of *H. pylori* after single-loop reconstruction due to the alkaline milieu, which is absent with Roux-en-Y reconstruction [9]. Child reconstruction with modified Braun enteroenterostomy leads to a reduced incidence of marginal ulcers [9-11].

There are several procedures described in the literature for marginal ulcer perforation. Simple closure, omental patch closure, resection of the duodenal or gastro-jejunal anastomosis, followed by Roux-en-Y reconstruction. We recommend proceeding with any of the above-mentioned on a case-by-case basis and maintaining a high suspicion index for diagnosing marginal ulcers with perforation. Prevention strategies like bilateral truncal vagotomy can be done for patients with a history of peptic ulceration during PD surgery itself [12].

## Conclusions

Marginal ulcer perforation post PD is currently underestimated and can be associated with serious complications like bleeding and perforation. It should not become a cause of mortality in long-term survivors. Hence, maintenance of PPI prophylaxis can be advised for patients who are smokers, alcoholics, or those who are on NSAIDs and have a prior history of peptic ulcer disease. Endoscopy surveillance for at-risk patients is also advisable, but currently, there is no defined protocol for endoscopic surveillance post PD. Adding a truncal vagotomy procedure at index surgery may be a prudent approach for patients with a prior history of peptic ulcer disease. But the routine addition of vagotomy may not be needed in this era of antisecretory therapy.
